# The National Medical Union

**Published:** 1914-01-03

**Authors:** 


### THE PROFESSION OF MEDICINE.

### The National Medical Union.

THE Provisional Federating Council of the National Medical Union have formulated the following policy—viz:

1. To maintain the freedom of the medical profession in its professional and personal relations with the public.
2. To support the public in maintaining a corresponding freedom and to safeguard the national health.
3. To oppose the present arrangements for medical benefits under the National Insurance Acts and to decline professional service under such arrangements.
4. To preserve an independent attitude with regard to party politics.

Since its inception at the Conference of the Non-Panel Associations, held on November 29, the National Medical Union has actively pushed forward the initial steps of organisation. New non-panel associations have been formed, and members in isolated positions in all parts of the country have been enrolled at headquarters.

In order that there shall be no misconception as to the objects of the National Medical Union, and in view of the crisis through which the medical profession is passing, it is necessary to make it clear that the Union is an organisation pledged solely to non-panel interests.

The Union is established in its offices in the *Morning Post* Buildings, 346 Strand, London, to which address all non-panel practitioners who are in sympathy with the above policy, and who have not already become members through their local non-panel association, should communicate.

### THE NON-PANEL MOVEMENT IN YORKSHIRE.

Organisation of non-panel practitioners is proceeding in Yorkshire, where the Huddersfield Non-Panel Association is calling a meeting at which federation with the National Medical Union will be proposed.

The Huddersfield Association is in touch with Halifax, Bradford, York, and Leeds. Non-panel practitioners in Yorkshire who desire to support the movement are requested to communicate with Dr. R. H. Trotter, Sunnybank, Holmfirth, Yorks., or to apply direct to the Hon. Secretaries of the National Medical Union, *Morning Post* Buildings, 346 Strand, London.

---

The policy formulated in the official announcement of the National Medical Union printed above is simple and deals with broad principles only, two essential merits in stating the intentions and outlines of work for any association with a real programme before it and a mind made up to tackle that programme.

The first article, and since it is placed foremost we may take it for granted that it stands foremost in the esteem of those who direct the National Medical Union, reads:—"To maintain the freedom of the medical profession in its professional and personal relations with the public." In bowing its neck to the yoke a year ago, the medical profession incurred the suspicion that it neither deserved nor desired its freedom, a thing in itself so incredible about a body of Englishmen, that it is hardly worth refuting but for the fact that the taunt has actually been levelled. Nevertheless, it is certain that the need of freedom, and the recognition of that need by the individual members of the medical profession, are much greater now than was the case a year ago; and this is one of the chief reasons for the increasing membership and influence of the non-panel bodies, now federated together as the National Medical Union.

On the second article of faith it is scarcely necessary to dwell, for it is the complement and corollary of the first one. Freedom for the medical profession in its relations with the public implies a corresponding freedom in the other party to the bargain; and the safeguarding of the national health, which is also mentioned in the article, is an aim which the medical profession has always at heart.

In the third article of faith the present administration of the medical benefits of the Insurance Act is condemned; and the main plank of the Union's programme is declared in the phrase "to decline professional service under such arrangements." This is commendably frank and clear, and no one who joins the Union can possibly have any misapprehension of the principles he is professing and espousing in doing so.

Last of all, the Union undertakes "to preserve an independent attitude with regard to party politics." Just at the moment, obviously enough, the Union is "agin the Government"; for the Chancellor of the Exchequer has deliberately made the Act and everything to do with it a partisan matter for the Radical Party. But it is highly probable that at some future time—and possibly sooner than at the moment would seem likely—National Insurance may be promoted to a higher and purer atmosphere than that of the party cockpit. And even if this desirable consummation fails to come about, the turn of the wheel of fate may bring the Government of the country into the hands of some other party, from which it would then become the duty of the Union to extract a satisfactory amendment of the provisions of the Act. So this provision for eliminating all party political considerations from the propaganda of the Union is unquestionably wise and prudent. It is hardly necessary, we suppose, to add that the removal of the Union's offices to premises in the same block as those of a well-known and influential Conservative daily paper has no significance whatever which would weaken in any way the declaration of political independence now made so emphatically by the Union.

### Dinner to Dr. Addison.

Amongst the most conspicuous of those to whose aid and countenance Mr. Lloyd George owed his victory over the medical profession a year ago was Dr. Christopher Addison, M.P. The medical profession may, therefore, be expected to note with some interest that a complimentary dinner is being promoted to him, and that the Chancellor is to take the chair. Two prominent supporters of the Ministerial Party are undertaking the arrangements, both of them doctors who have been knighted for semi-political services. It is, no doubt, quite natural that Mr. Lloyd George should feel grateful to those who placed their party politics before the interests of their professional brethren; but it is questionable whether to exult over the vanquished so ostentatiously is diplomatic. Dr. Addison may be a good lobbyist, but the medical profession have not forgotten that this teacher of anatomy did much to rivet the yoke of servitude upon their necks, and the reminder will serve to keep that recollection vivid longer than might otherwise have been the case. No one can reasonably blame Dr. Addison for his course of action; he was elected to Parliament because he was a Ministerialist, not because he was a doctor; and in putting his politics before his profession he was doing his best to advance his own interests. It does, however, seem odd that this should be considered so rare and unusual a course as to demand a complimentary dinner in celebration of it. Surely a decoration or an office would have been sufficient!

### Combination and Character.

In the letter which we print on p. 379 Mr. Nicholls asks some pertinent questions of a kind such as many clear-headed practitioners must have asked themselves in reviewing the situation. There is, however, in our opinion, a satisfactory answer to these interrogations. Admittedly the failure of a year ago was due more to a failure in moral courage and character than in combination and organisation, though in the latter respects also there was great shortcoming. Admittedly, without a retrieval of courage and character in the future, the outlook for the medical profession is gloomy. Admittedly, as Mr. Nicholls puts it, "individuality, self-respect, and independence" are necessary to keep the profession of medicine afloat. Without them associations and unions are useless: that is agreed, and is surely obvious. But without organisation these qualities are equally helpless. The two things are equally necessary and mutually dependent; and this, we think, is the true answer to Mr. Nicholls' very ably expressed question.

